# Endoscopy in surgery

**DOI:** 10.3389/fgstr.2023.1186945

**Published:** 2023-11-20

**Authors:** María Rita Rodríguez-Luna, Silvana Perretta

**Affiliations:** ^1^Surgical Research Department, Research Institute against Digestive Cancer (IRCAD), Strasbourg, France; ^2^ICube Laboratory, Photonics Instrumentation for Health, Strasbourg, France; ^3^Department of Digestive and Endocrine Surgery, University of Strasbourg, Strasbourg, France; ^4^Institut de chirurgie guidée par l'image (IHU)-Strasbourg, Institute of Image-Guided Surgery, Strasbourg, France

**Keywords:** flexible endoscopy, digestive surgery, endoscopic salvage, clinical outcomes, innovation, endoscopic training curricula

## Abstract

The expanding role of flexible endoscopy (FE) has helped to establish better diagnostic strategies and fewer invasive therapies within the lumen of the gastrointestinal (GI) tract. Endoscopic skills represent critical tools for surgeons since they markedly impact perioperative outcomes. Although it is widely recognized that endoscopy plays a key role in digestive surgery, endoscopic curricula and syllabi may vary depending on geographical regions, which have their own standardized guidelines such as the United States and countries with numerous disparities such as Western Europe. Such heterogeneous practices represent a call for action, particularly as surgical societies aim to expand cutting-edge endoscopy within surgery. This article outlines the crucial role of intraoperative endoscopy in commonly performed digestive surgeries and stresses the need to develop standardized endoscopic training curricula in surgery, particularly in Europe.

## 1 Introduction

Surgeons largely contributed and fostered the advent of both diagnostic and interventional endoscopy as they have a deep understanding of GI anatomy and physiology. Examples of such developments include colonoscopy, polypectomy, endoscopic retrograde cholangiopancreatography (ERCP), biliary stenting, and percutaneous endoscopic gastrostomy (PEG) (1, 2). In the past 30 years, surgeons have also brought sophisticated endoscopic techniques to the submucosal space, such as endoscopic submucosal dissection (ESD) and peroral endoscopic myotomy (POEM) (3). These endoscopic modalities have been referred to as third-space endoscopy and have broadened endoscopic capabilities for drug delivery, muscle and nerve biopsies, removal of intramural lesions, tissue harvesting (i.e., stem cells), and monitoring device placement (4).

In addition, the less invasive nature of interventional endoscopy could potentially replace its laparoscopic counterpart in many GI conditions. The Japanese experience is a clear example in which endoscopic treatment of early gastric cancer (EGC) is surpassing surgery and is the recommended approach in cT1a differentiated-type carcinomas of less than 2cm without ulceration (5). ESD indications are rapidly expanding worldwide since similar oncological outcomes are being reported in experienced hands (ESD >1,000) (6), with less morbidity and a better quality of life (QoL) compared to surgery.

Endoscopic sleeve gastroplasty (ESG) is another example of an endoscopic alternative to surgery. An organ-sparing procedure based on gastric tubulization and shortening using multiple full-thickness non-absorbable sutures, ESG has proven to be safe and effective for weight loss and comorbidity resolution (7). Even though bariatric surgery has been considered the gold standard treatment in morbidly obese patients, only 1% of patients undergo surgery because of limited access, patient preference, risks, and healthcare expenses (8, 9). Consequently, ESG has the potential to reduce such gaps, hence reaching a larger proportion of morbidly obese patients. In addition, endoscopic options for weight loss can target populations with obesity that are not candidate for surgery.

**Figure 1:**
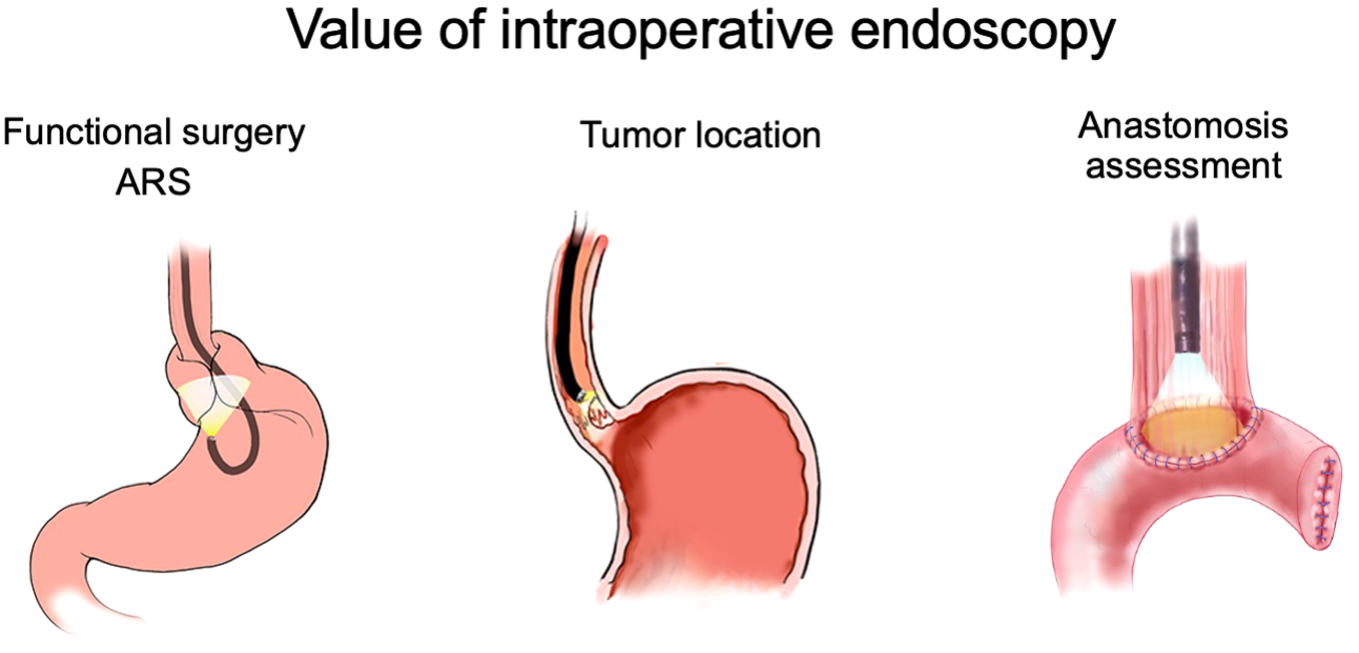
Overview of the value of intraoperative endoscopy during different GI surgeries.

The rapid evolution of surgery calls for adequate training and proficiency in endoscopic technologies. This article aims to address the importance and relevance of endoscopic proficiency in functional surgery, oncologic resections, and collaborative endo-laparoscopic techniques (**Figure 1**). Endoscopic curricula and syllabi are also highlighted, along with future perspectives.

## 2 Functional surgery

Functional diseases of the upper GI tract include pathologies associated with motility disorders (i.e., achalasia, specific types of esophageal diverticula (i.e., pulsion diverticula), and gastroesophageal reflux disease [GERD])which often have a significant impact on patient QoL. In such benign conditions, surgical therapies necessitate excellent clinical outcomes focusing on functional improvement while avoiding complications.

### 2.1 Achalasia

Achalasia is an esophageal disease with impaired relaxation of the lower esophageal sphincter (LES) and aperistalsis (10). Endoscopic treatment modalities include endoscopy-based injection of botulinum toxin, endoscopic pneumatic dilation, and POEM. POEM represents cutting-edge “third-space endoscopy”. Although it has been extensively accepted thanks to its long-term safety and efficacy, it requires a significant learning curve and it is not applicable to all achalasia patients (11–13). Flexible endoscopy (FE) could also be helpful during a laparoscopic Heller myotomy procedure, still performed in many centers (14). FE allows surgeons to clearly identify the gastroesophageal junction, guaranteeing adequate myotomy length on the gastric side, helping to prevent any inadvertent mucosal perforation and to assess the aspect and position of either the anterior Dor or posterior Toupet fundoplication (15). In addition, the use of FE also facilitates the deployment of endoscopic devices such as the EndoFLIP™ impedance manometry system (Medtronic) to assess minimum diameter (Dmin), cross-sectional area (CSA), and distensibility using impedance planimetry before and after myotomy (16). Post-myotomy objective measurements could better ensure symptom resolution (17).

### 2.2 Esophageal diverticula

Intraoperative FE may be helpful when treating esophageal diverticula by allowing to precisely identify the location and dimensions of the diverticulum. It also helps to achieve a complete diverticulectomy during diverticulum stapling, while ensuring that the stapling does not compromise the esophageal lumen, and allows to facilitate mucosal preservation during myotomy.

**Figure 2:**
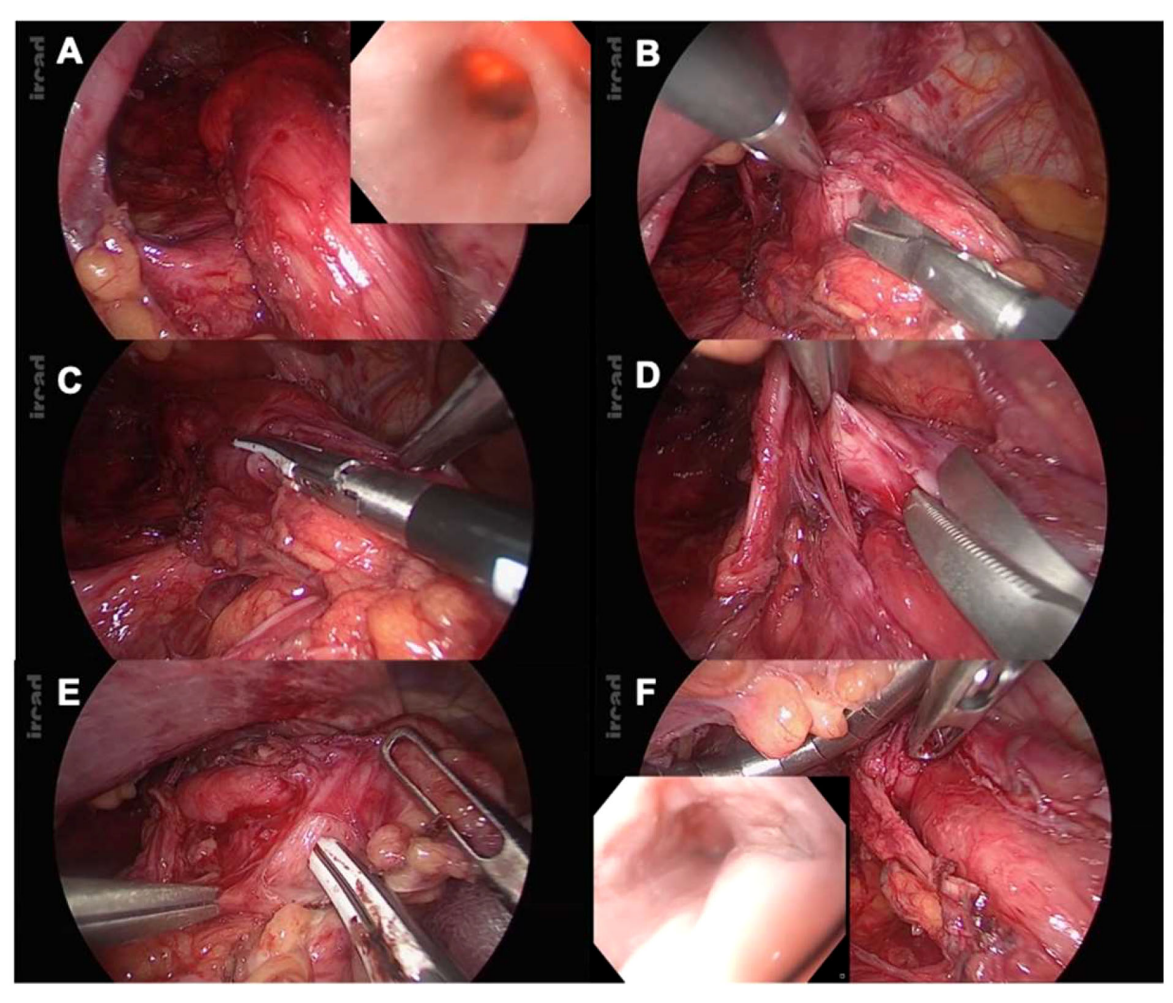
**(A)** Epiphrenic diverticula identification: laparoscopic and endoscopic views; **(B)** Starting point of the myotomy at the right side of the esophagogastric junction (EGJ); **(C)** Proximal myotomy extension with vessel-sealing device (LigaSure™, Medtronic); **(D)** Myotomy at the level of the epiphrenic diverticula: cold dissection is used, hence preventing any mucosal perforation; **(E)** Myotomy extension 2cm from the gastric wall; **(F)** Intraoperative FE is used to rule out perforation and assess stenotic release.

Non-resective management with simple myotomy is an acceptable option in small esophageal epiphrenic diverticula (**Figure 2**).

### 2.3 Gastroesophageal reflux disease

Gastroesophageal reflux disease (GERD) is highly prevalent in Western countries (18). Treatment options include medical therapy, surgery, and endoscopy. Of note, over the last decade, endoscopic therapies have expanded, including non-ablative radiofrequency (RF) treatment, Stretta therapy (Restech, Houston, TX), which uses four nitinol needle electrodes to deliver pure sine-wave energy on the muscular layer of the esophageal wall, and more particularly so on the LES and the gastric cardia, in order to relieve reflux symptoms (19).

Radiofrequency has also been used to treat GERD complications such as Barrett’s esophagus (BE). In this scenario, ablative RF is used to safely eradicate dysplastic BE while reducing the risk of metachronous esophageal adenocarcinoma. Radiofrequency ablation (RFA) is applied through a circumferential balloon or focal ablation probe on the flat BE’s mucosa for thermal eradication of dysplastic tissue, allowing for normal squamous epithelium regrowth. Available evidence from the AIM dysplasia trial, the SURF study, and multiple pooled analyses have led RFA to be considered as the first-line therapy for BE ablation (20).

Transoral incisionless fundoplication (TIF 2.0) is another relatively new endoscopic technique to treat GERD. It consists in the creation of an endoluminal esophagogastric fundoplication in patients with relatively normal anatomy (HH less than 2cm) (21). The TIF 2.0 EsophyX vs. Medical PPI Open label (TEMPO) trial, which is a multicenter, controlled, randomized study, has shown promising results with 86% of symptoms resolution at five years of follow-up (22). Although there is encouraging evidence for this innovative device, the recent Multi-Society Consensus Conference and Guideline on the Treatment of GERD suggests that adult patients with GERD may benefit from TIF 2.0 over continued PPI therapy (conditional recommendation with moderate certainty of evidence) (23). Undoubtedly, endoscopic therapies have broadened the surgeon’s armamentarium to treat some GERD cases.

Surgeons treating patients with GERD should have a thorough understanding of the underlying pathophysiology, critical steps, and pitfalls to ensure reliable long-term functional outcomes. Laparoscopic fundoplication includes critical steps in which FE guidance helps to obtain objective endoluminal assessment as follows: a) confirmation of the Z-line location; b) assistance during esophageal lengthening procedures; c) identification of anatomical landmarks in complex cases; d) assessment of valve tightness; e) endoscopic mucosal fold appearance on retroflexion.

**Figure 3:**
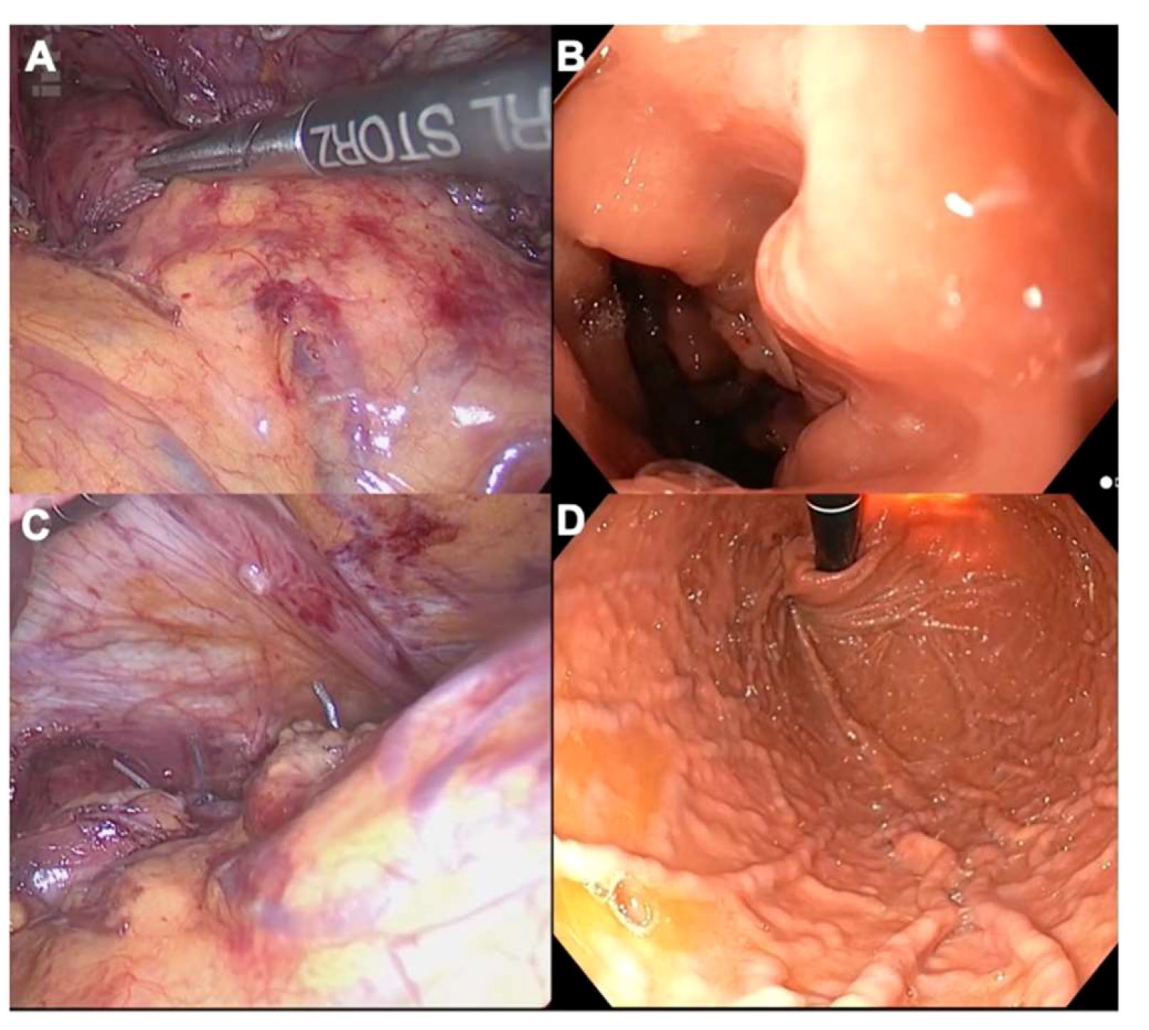
**(A)** Laparoscopic identification of the Z-line during an anti-reflux surgery; **(B)** Endoscopic counterpart; **(C)** Laparoscopic views of a Nissen fundoplication in retroflexion; **(D)** Endoscopic counterpart. Please note that the stomach must be completely insufflated with carbon dioxide to assess the symmetry of the gastric folds in the fundoplication.

The length of intra-abdominal esophagus plays a critical role in anti-reflux procedures. A total length of 2cm is necessary to preclude axial tension and subsequent recurrence. As mentioned previously, intraoperative endoscopy helps to accurately distinguish the Z-line (**Figure 3**). Notably, Chang et al. have shown that, when comparing endoscopic and laparoscopic-based judgment in up to 10% of GERD cases, endoscopy helped to position the Z-line higher. This pertinent piece of research stresses the need for endoscopic assessment during anti-reflux surgery, since the Z-line location is strongly correlated with wrap position, and as such, FE may preclude “slippage” (24). In cases where 2cm of intra-abdominal esophagus are not reached after intramediastinal dissection, esophageal lengthening procedures (e.g., Collis-Nissen gastroplasty) represent a valid option. The neoesophagus is obtained by means of a wedge resection of the proximal stomach. FE helps to check staple line integrity and perform a leak test, preventing major complications such as leaks.

By definition, redo cases for valve slippage, intrathoracic migration, and mesh erosion are more complex with higher complication rates, and FE represents a valuable tool when anatomy is disrupted and anatomical structures are difficult to identify (25). Endoscopic real-time imaging during intramediastinal dissection helps to accurately distinguish postoperative fibrotic tissue, thereby maintaining the dissection planes in a “safe zone” and preventing lesions to critical structures such as nerves, pulmonary veins, etc. In cases of mesh-related complications such as migration, esophageal erosion or strictures, a combined endoluminal and laparoscopic assessment during mesh removal helps to preserve mucosal integrity.

**Figure 4:**
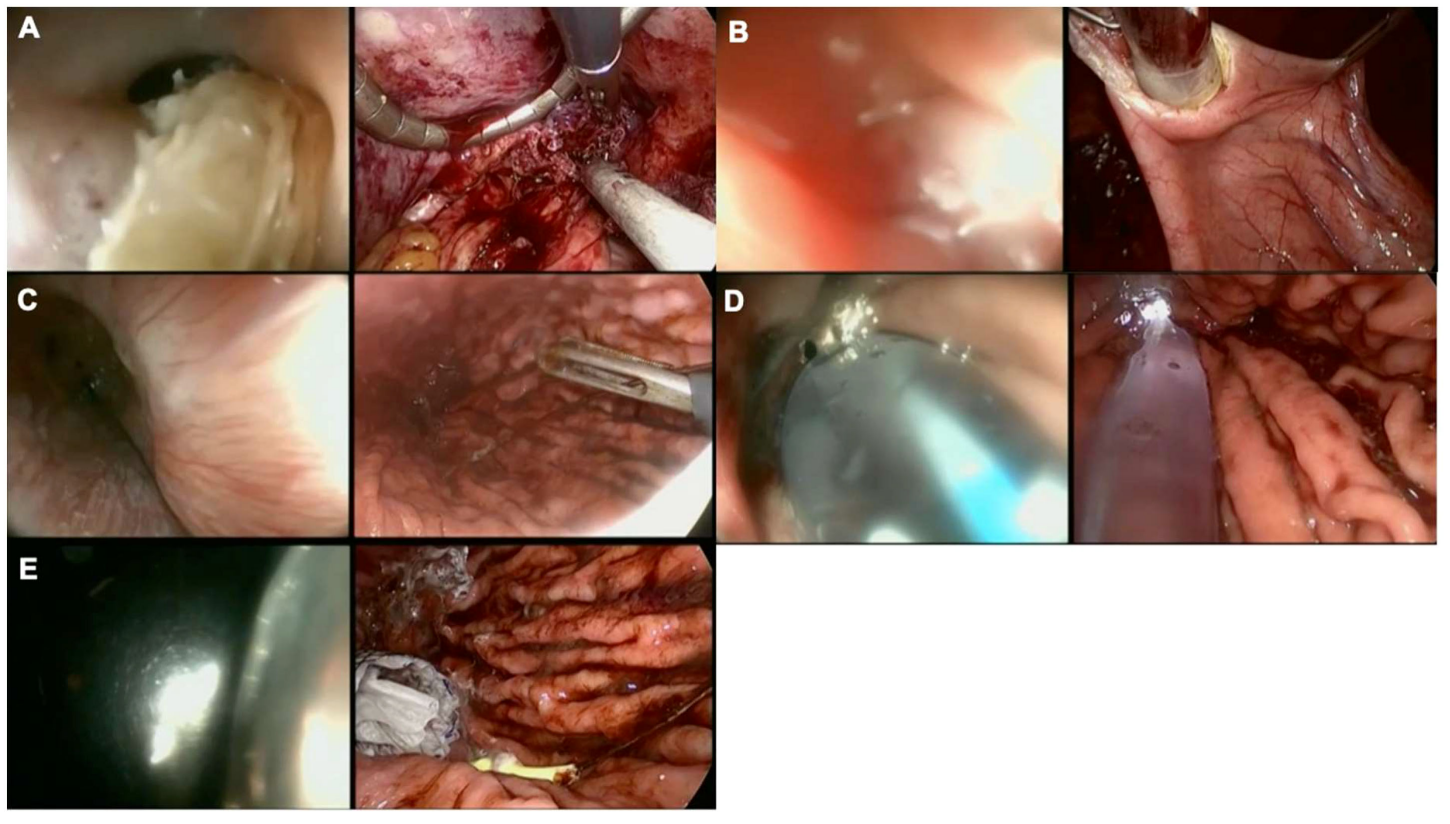
Collaborative endo-laparoscopic surgery after anti-reflux operation and keyhole mesh erosion. Left images represent endoscopic views while right images show laparoscopic views. **(A)** Mesh identification, endoscopic and laparoscopic mesh removal, the endoscope cannot go through the EGJ junction, a guide wire is endoscopically delivered; **(B)** Intragastric surgery to retrieve the guide wire placed endoscopically; **(C)** Control of the distal end of the guide wire; **(D)** Balloon dilation; **(E)** Ultraflex™ (Boston Scientific, United States) self-expandable metal stent (SEMS) placement.

Finally, in severe cases where mesh fibrosis and/or erosion leads to a complete loss of anatomy, the combination of FE, laparoscopy, and even transgastric surgery in some cases, enable hybrid approaches such as on-table dilatation and esophageal stent placement (**Figure 4**). In such very challenging cases, a collaboration between endoscopic and laparoscopic skills supports organ-sparing procedures, essential when treating benign diseases.

## 3 Intraoperative tumor detection

Esophagogastric junction (EGJ) tumors truly emphasize the need for precise tumor location in foregut surgery. Although surgical strategies for EGJ Siewert types I and III tumors have been well-defined, the treatment of Siewert type II tumors has changed over time since these tumors have been considered to be either esophageal or gastric carcinomas (26). The repertoire of surgical options includes esophagectomy or extended gastrectomy with transhiatal distal esophageal resection (27). Consequently, FE supports decision-making by determining the tumor location at the epicenter or from the majority of tumor masses. Additionally, intraoperative endoscopic assessment enables real-time evaluation of proximal tumor extent ensuring at least a 2cm distance from the tumor margin for R0 resections (28).

Similarly to EGJ tumors, the localization of colorectal tumors is generally performed prior to surgery. However, current diagnostic modalities, including mucosal tattooing in colonoscopy and computed tomography with 3D reconstruction, are insufficient. A recent publication found that on-table endoscopy changed the surgical planning in up to 16.7% of patients, and more precisely in patients with tumors located in the transverse or sigmoid colon, stressing the need for on-table endoscopic availability (29). Cerdán Santacruz et al. have recently stressed the importance of intraoperative colonoscopy in patients with incomplete preoperative colonoscopy, changing attitudes in 5% of patients with 2% of the global sample corresponding to the finding of synchronous tumors. Interestingly, up to 42% of intraoperative colonoscopies were performed transanastomotically without any postoperative complication (30).

## 4 Anastomotic assessment

After resective or bariatric surgeries, the restoration of GI continuity is performed using either manual or stapled anastomosis. Evaluation of the mucosal surface may help to identify and treat bleeding and anastomotic leakage. Significantly fewer complications have been reported in patients who undergo gastrectomy when FE is used (3.4 *vs.* 8.9%; p <0.01) (31). In addition, in case of anastomotic concerns, preventive self-expandable metallic stents (SEMS) can be intraoperatively delivered with endoscopic devices to protect the anastomosis, allowing for patient-based tailored approaches when handling complex cases (32). Similar principles can be applied when assessing low GI anastomosis.

In a cohort of 338 patients, Shamiyeh et al. found that an intraoperative endoscopic evaluation of circular-stapled colorectal anastomosis could well detect early anastomotic bleeding and leakage (33). Although promising results have also been published regarding the use of intraoperative endoscopy during colorectal resections, there is still no standardized practice. Endoscopic anastomotic assessment depends on the surgeon’s endoscopic proficiency, highlighting the need for training and endoscopic implementation in surgery (34).

## 5 Laparo-endoscopic collaborative surgery

**Figure 5:**
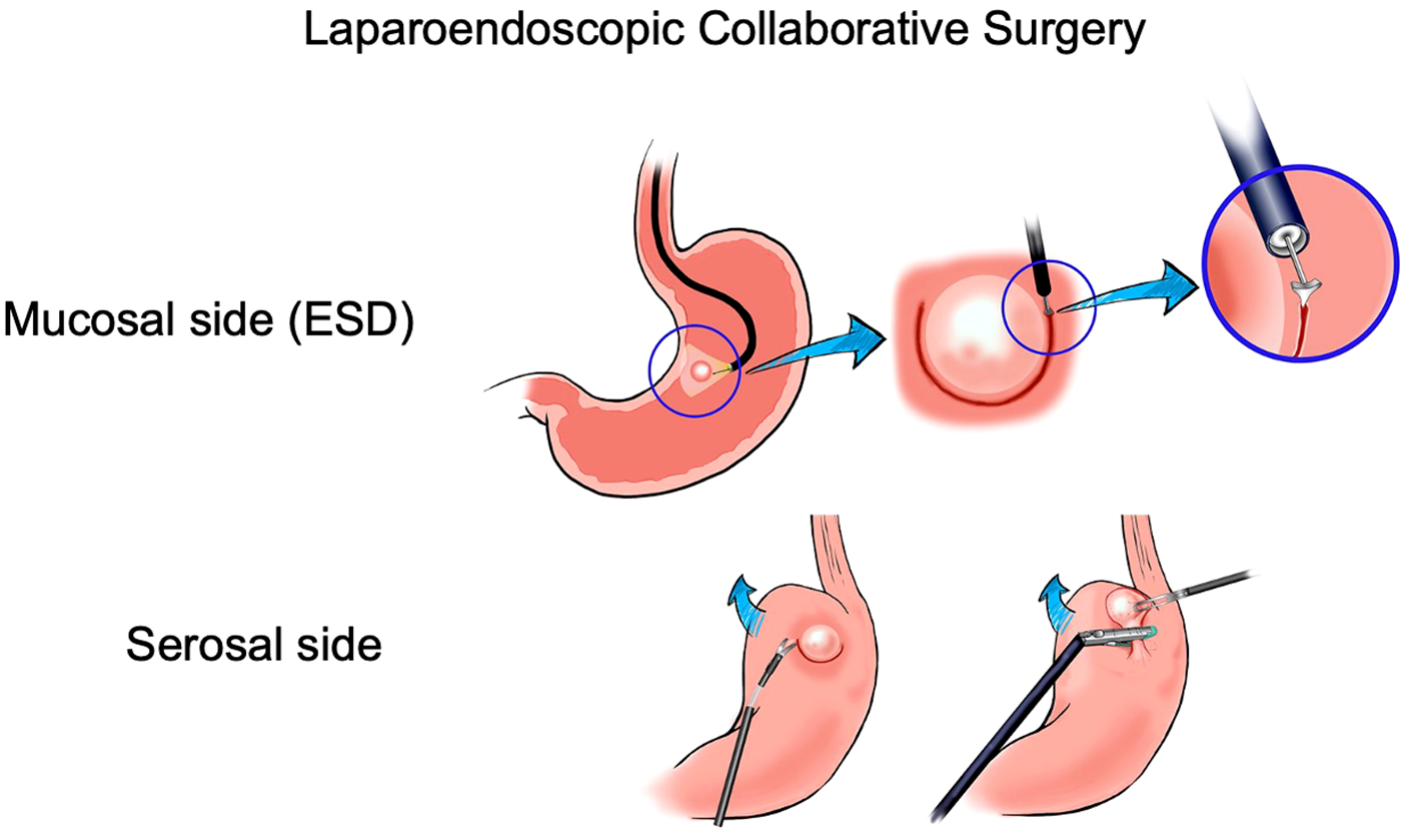
Overview of key technical steps in laparoendoscopic collaborative surgery.

Laparo-endoscopic procedures are collaborative, developed to overcome the limitations of purely endoscopic techniques such as ESD and support oncological outcomes while sparing tissue and preserving physiology. Laparo-endoscopic collaborative surgery (LECS) was first described in 2008 with the objective of providing precise oncological therapy in gastrointestinal stromal tumors (GISTs), where precise tumor location and surgical therapy are complex, and with purely laparoscopic techniques such as wedge resection. The steps in LECS described by Hiki include the following: (a) determination of an accurate incision line from the endoscopic view; (b) ESD around the tumor; (c) artificial perforation of the stomach using an endoscopic device along the resection line; (d) seromuscular dissection using an endoscopic or laparoscopic device; (e) removal of the tumor from the abdominal cavity; and (f) closure of the laparoscopically opened gastric wall with hand-sewn sutures or a stapling device (35) (**Figure 5**). This technique was validated in multiple clinical trials showing good oncological and functional outcomes. Laparo-endoscopic collaborative surgery has been extended to the duodenum and colon, known as laparoscopy and endoscopy cooperative surgery for colorectal tumors (LECS-CR) (36, 37).

## 6 Management of complications

The endoscopic treatment of surgical complications constitutes tangible progress in digestive surgery, promoting their endoluminal management and avoiding reoperations as much as possible. Endoscopic rescue has been vastly explored after bariatric surgery, and it has paved the way for treating esophageal, gastric, and colorectal surgery complications (38). In bariatrics, the incidence of leak and fistula ranges from 0 to 7% and from 1 to 7% in laparoscopic sleeve gastrectomy (LSG) and from 0.1 to 8.3% and from 1 to 5% in Roux-en-Y gastric bypass (RGYB), respectively. Such complications represent the greatest concern due to increased patient morbidity and mortality (39–42). Endotherapy has been successfully used to treat acute and chronic leaks occurring after both LSG and RYGB.

Simple devices such as pigtails, once used to drain pancreatic pseudocysts, have taken a pivotal role in the therapeutic algorithm of digestive surgery, thanks to their high efficacy and low cost (43, 44). Pigtails have been applied to bariatrics in order to induce internal drainage and promote granulation tissue formation. Manos et al. have shown that successful endoscopic treatment of LSG leaks using pigtails may be achieved in an average of 3.2 months with 2.8 endoscopic interventions (45). Additionally, in such patients, FE helps to identify and treat anatomical abnormalities that are closely related to the etiology of leaks such as stenosis, twists, and flow obstruction. Balloon dilation can be performed as part of the first-line treatment.

Endoscopic rescue has become more feasible thanks to the vast array of commercially available devices including luminal self-expanded metal stents (SEMS) (46), which can be partially or fully covered. The latest European Society of Gastrointestinal Endoscopy (ESGE) guidelines recommend temporary stent placement to treat esophageal leaks, fistulas, and perforations (47). In bariatric leaks, SEMS can also be used in combination with pigtail drainage (43). Stent migration is one of the most common adverse events after SEMS placement, requiring close clinical follow-up (48) and stent fixation.

Endoscopic over-the-scope-clip (OTSC) systems are innovative in endoluminal therapy. This relatively new class of endoclips enables proper tissue capture and apposition. OTSC systems represent a successful approach in well-selected patients with postoperative acute LSG leaks. A systematic review of 10 pooled studies showed an overall successful closure of 86.3% (49). Another meta-analysis showed that clinical success decreases when comparing acute to chronic leaks, with a mean of 81.4% [95% CI: 77.0-85.3] and a mean of 63.0% [95% CI: 53.0-72.3], respectively (50). OTSCs can also be used to handle gastrointestinal hemorrhage with a clinical success of 87.5% [95% CI: 80.5-93.2] (50–52). In our practice, we have also used OTSCs to fix SEMS proximally, thereby preventing stent migration.

Endoscopic suturing devices and vacuum-assisted closure (VAC) systems have broad endoscopic capabilities for treating gastrointestinal wall defects (53). Endoscopic suturing devices, such as the OverStitch™ (Apollo Endosurgery, TX, United States), enable full-thickness suturing and have been used to treat a wide variety of GI fistulas. In one of the largest series of endoscopic suturing used to manage fistulas (51.8% of gastrogastric fistulas), an immediate success rate of 100% was only sustained for nearly 40% of patients (54). Despite the high success rate, endoluminal suturing is costly and technically challenging, requiring a thorough understanding of the devices and appropriate training.

Endoluminal vacuum therapy is another option consisting of an open-pore polyurethane sponge (EsoSPONGE^®^, B. Braun Melsungen AG, Melsungen, Germany) placed endoluminally or endocavitarily. The sponge has a 100cm drainage tube connected to a negative pressure system, which allows for continuous drainage while promoting granulation and tissue healing (55). It has been used to treat leakage after esophageal and rectal surgery. In upper GI surgery, vacuum therapy has been primarily used to treat leaks, as well as in rescue therapy. In a retrospective multicentric study from Da Hyun et al., clinical success was found in 70.6% of cases in 119 patients, and most of them received vacuum therapy as a primary treatment (89 patients) (56). Yang et al. showed that vacuum therapy requires a median of 14.5 days of endoluminal vacuum (EVAC) therapy and a median of 5 interventions (57). Current evidence originating from two meta-analyses suggests that, as compared to SEMS, EVAC is significantly associated with a higher rate of leak closure (58, 59).

In colorectal surgery, one of the latest systematic reviews with 17 pooled studies (276 patients) has shown a success rate of 85.3% using EVAC (60). The main disadvantage of EVAC is the need for sponge changing every three to four days.

Over the past 20 years, these innovative endoscopic technologies have expanded endoluminal treatment options and will undoubtedly pave the way for future treatment modalities. Surgeons who are daily confronted with adverse events should move the limits of current therapeutic strategies forward.

## 7 Endoscopic training curricula in surgery

As highlighted in this article, there is a clear role for surgeons to promote endoscopic innovation since they are confronted with surgical scenarios with unmet needs on a daily basis. Since 2014, the American Board of Surgery (ABS) and the Society of American Gastrointestinal and Endoscopic Surgeons (SAGES) have been committed to establishing reliable methods and criteria to assess endoscopic psychomotor skills and competencies (61). Endoscopic skills have been more recently considered part of required general surgery curricula and syllabi in Western European countries; yet, clinical practice remains limited as curricula differs from one country to another, and no consensus has been met (62). Although interventional gastroenterologists and surgeons in Sweden and Poland have been sharing endoscopic activities almost equally, endoscopic practice is mostly undertaken by gastroenterologists in France, Belgium, and in the Netherlands (63).

Importantly, since 1994, the Joint Association Group (JAG) on GI endoscopy in the UK set up a task force to ensure endoscopic standards. In 2009, an online JAG Endoscopic Training System (JETS) portfolio was developed to improve access to and quality of endoscopic training, as demonstrated by the following website: https://jets.thejag.org.uk/Home. JETS aims to standardize endoscopic practice and training across the UK, providing certification to those who demonstrate competence. Over 650 certificates are issued annually. However, only up to 30% of these certificates are issued to GI surgeons. Most recent UK surveys continue to demonstrate disparities between surgical and gastroenterology trainees (64).

Overcoming obstacles to endoscopic practice and training among general surgeons represents a critical concern. As previously emphasized, intraoperative endoscopy plays a key role in every step of the surgical care, from diagnosis and treatment to intraoperative guidance, supporting decision-making processes, and preventing and handling complications. As a result, restricted endoscopic practice may negatively impact surgical patient outcomes. To promote high-quality surgical endoscopy and guarantee the surgeons’ right to practice flexible endoscopy throughout European countries, the European Association for Endoscopic Surgery (EAES) has recently created a Flexible Endoscopy Subcommittee within the Technology Committee. In a recent survey from the EAES that gathered more than 1,500 participants (unpublished data), a high heterogeneity was found in mandatory endoscopic training and practice as part of surgical curricula. There is a need to clearly understand the reason for such discrepancies, including regional healthcare policies, interest, and training availability to provide meaningful task force aiming at standardized practice. The survey found that a significant number of surgeons considered that performing flexible endoscopy trespass the territory of interventional gastroenterologists. Undoubtedly, this misperception should change and promote the close collaboration between interventional gastroenterologists who have a vast experience in the endoscopic management of GI diseases and surgeons. Joint efforts can improve clinical practice and innovation in the field of “endoscopic surgery”.

As a result, synergism between these two subspecialities may allow for the development of more sophisticated endoscopic units and operative rooms equipped for endoscopic surgery since a close surveillance on quality indicators including facilities and equipment, endoscopic procedures, and outcome measures are required to stablished competence (65).

Surgeons should follow structured training curricula in endoscopy in which core factors are continuously evaluated to provide the highest quality standards. As suggested by Siau Kethi et al., program-based trainings should focus on the trainee, the trainer, and the training program since a structured comprehensive curriculum has demonstrated better outcomes as compared to self-regulated learning (66, 67).

Regarding trainees, there is still little evidence showing how inherent abilities and developed laparoscopic psychomotor skills improve endoscopic training. However, when assessing EGD and colonoscopy competency, surgical training has been associated with the highest multivariate odds ratios for independent task completion (67, 68). Highlighting differences between gastroenterologists and GI surgeons at early-stage endoscopic training stresses the need for specific endoscopic curricula.

Defining competence benchmarks also represents a major benefit of training standardization and continuous performance tracking over time. Virtual reality (VR) simulators such as the Simbionix GI Mentor VR computer simulator (Beit Golan, Israel) have played a key role in educating novice trainees at an early stage, as highlighted by the latest systematic reviews which demonstrate their advantages (69, 70). Interestingly, a randomized control trial (RCT) found that trainees with 10 hours of unsupervised VR training presented higher objective competency rates during bedside training as compared to those without VR training (71). However, shortcomings of VR (e.g., low level of realism, no haptic feedback, and high cost maintenance) have been identified, precluding its widespread use.

With the aim of expanding endoscopic skills for surgical residents and novices, the basic endoscopic skills training (BEST) box was created and validated using the Global Assessment of Gastrointestinal Endoscopic Skills Upper Endoscopy (GAGES-UE) scoring system. The BEST box consists of reusable endoscopic material to teach basic tasks, including forward peg transfer, retroflexion peg transfer, puncturing, snaring, clipping, and cannulation (72). In our practice, the BEST box represents the first contact in all endoscopy training courses. The recent non-inferiority RCT which compared VR to BEST box training showed comparable results, suggesting that low-cost endoscopic box trainers could help to democratize flexible endoscopic curricula (73).

For hands-on training in advanced therapeutic procedures (e.g., ESD, POEM, ERCP, ESG), *ex vivo* and *in vivo* animal models represent a good strategy prior to safe patient-based training. Finally, certification in surgical endoscopy should be pursued to standardize endoscopic practice in European countries.

## 7 Conclusions

Endoscopy represents a set of skills, which undoubtedly enhance surgical practice, providing more precision and promoting organ-sparing, function-preserving procedures. Therapeutic endoscopic capabilities are swiftly expanding, marking a shift in the standard of care for many GI digestive diseases. The key role of endoscopy is reflected in the mandatory and standardized curricula available in the United States and Canada, which has highlighted the need to improve training capabilities and to define standardized high-quality education among surgeons in European countries. Surgery is undergoing major transformations with a clear need for an endoscopic workforce to improve patient outcomes through standardized practice. Surgeons must embrace their endoscopic heritage and acknowledge the use of innovative technologies such as endoscopic robots engineered to overcome the current limitations of flexible endoscopes. Endoscopic robotic systems can replicate surgical tasks such as triangulation, tissue manipulation, dissection, and apposition within the lumen. The positive impact of endoscopic robots on the learning curve and decreased inter-variability among operators, together with the enhanced diagnostic power of smart imaging systems, may help to democratize endoluminal and transluminal organ-sparing therapies in the near future.

## Data availability statement

The original contributions presented in the study are included in the article/supplementary material. Further inquiries can be directed to the corresponding author.

## Author contributions

The authors confirm contribution to the paper as follows: study conception and design: RR and SP. Data collection: RR. Analysis and interpretation: RR and SP. Draft manuscript preparation: RR and SP. All authors contributed to the article and approved the submitted version.
